# Random Notes on Simplicity of Fracture-Apparatus

**Published:** 1881-01-20

**Authors:** William H. Morse


### RANDOM NOTES ON SIMPLICITY OF FRACTURE-APPARATUS.

BY WILLIAM H. MORSE, M.D.

One of the most patent axioms of surgery is that fracture-apparatus shall be such as will the best assist nature in procuring reunion of osseous integrity and in preventing deformity. The idea has obtained that it makes little or no difference what apparatus is used if it is of a character that will serve the ends for which it is employed. Out of this idea others corollary to it have been framed, until the opinion prevails that everything, from the bandages to the bed, must have some certain elaboration. As a consequence, the market is full of patented apparatus, beautiful in theory and often excellent in principle; and every surgeon has some favorite splint or plaster, or exerts himself to invent one.

It was my privilege in my student days to read the works of Gross, Hamilton, Erichsen, Bryant and others, each discussing different apparatus, all elaborate and some complicated. At the same time I had the pleasure of receiving the teachings of Dr. John Swinburne, of Albany, who, as all know, is an apostle of simplicity in fracture-apparatus. In the text-books I read of many vexed modes of treatment, and from Prof. Swinbuurne's lips I heard the advocacy of simplicity. Out of college and in practice I found that it was impossible to serve two masters. Bewilderment came with my first fracture-case, and I found it difficult to make a choice from among many splints and many modes of treatment. Like all other physicians, I had to learn that the instructions of text-books are good in theory, but all too apt to be poor in practice.

I chose to adopt simple treatment of fractures as easier to carry out and as serving the best purpose, and not because I dissented from the ideas of the text-books, or because of any disparagement of the intentions of authorities on the subject. Nature is in love with simplicity. The theoretical horticulturist may graft a tree, and confine the scion with patented appliances, and still not have the success that attends the labor of the farmer's boy, who, with string and grafting-wax, ties in and fastens the scion. The gardener, with patented and well-tried fertilizers, cannot always insure success in transplanting his plants. Nature prefers to name her own abettors. Yet no rational physician will condemn all fracture-apparatus. There are both good and bad to select from, but all of them are to be "well shaken before taken!"

What is simplicity in fracture-apparatus? The interpretation may signify one thing to one mind and something totally dissimilar to another. Extemporaneous appliances come the nearest to rigid simplicity. Splints that will the best keep the ends of the bone in connection, with simple arrangement of the bed and the principles of extension, are the essentials asked.

We know by the acquaintance of the text-books that there are the appliances of Hamilton, of Buck, and of others, and the physician trembles with awe of them, as does the boy who begins the study of Latin. To understand elaboration requires experience; to understand simplicity, unprejudiced discrimination is the only requisite. If simple methods of treatment will do that which is claimed for elaborate methods, and do it better, the physician can but exercise partial choice.

Notes of some cases, the treatment of which illustrates the employment of the principles of simplicity, will better give the meaning that I wish to show than mere detail can do.

CASE I.—*Fracture of the Femur.* Ann A. M., servant girl; age 27; in good health. Fell through a trap-doorway a distance of seven feet. Left femur fractured at the superior fourth; displacement considerable.

*Treatment.* Had a good mattress bed prepared; took a sheet for a perineal belt, folding it to a diameter of two inches; provided adhesive strips two inches broad; applied these spirally along both outside and inside of the leg, not over one another, but side by side, so as to obtain equal tension. At the foot a strip was doubled so as to provide a loop under the sole of the foot. These strips were fastened down by a roller, but on the occasion of the second dressing short adhesive strips were used in its place. The belt was secured to the head of the bedstead, and counter-extension was obtained by means of a cord passed through the loop of plasters beneath the sole of the foot and fastened to the foot of the bedstead. No splints were used, and the muscles and fascia took their place kindly. A bag of sand was placed at the foot to keep it from everting. The first extension was slight, but after ten days it was extended by degrees to its normal length. Union occurred nicely, and extension was continued for forty-one days. There was no appreciable shortening.

This is the "Swinburne method." There were no bandages or splints to embarrass the circulation, and besides affording perfect cleanliness, it was so that I could measure the limb daily. Dr. Swinburne has employed this method in cases much more complicated, where its advantages are much more conspicuous.

The foot of the bed is not raised, and no weights for greater degree of counter-extension are used. Yet in intra-capsular fracture these means are employed, and the resultant shortening should not be more than three-fourths of an inch.

CASE II.—*Fracture of both Tibia and Fibula.* A boy aged nine fell on the ice and fractured both bones of the leg.

*Treatment.* Provided a long and delicate splint (thirty inches long, two wide). To this attached a foot-piece, and in the upper end of the splint bored several holes. By the way, the splint was part of a clap-board ripped off the side of the house, and the foot-piece came off of the same board! The splint and foot-piece were secured to the outside of the limb by adhesive strips. Other strips were looped about the limb just below the knee, the loop touching the lower border of the patella on the anterior side. A cord was then passed through the loop, and carried thence through one of the holes in the splint above the knee-joint. Thus suitable extension was provided for. At intervals of five inches adhesive strips were placed around the leg to keep it in place on the splint. There was no great inflammation, else I should not have applied the roller strips until it had subsided. There was no self-displacement.

CASE III.—*Fracture of the Humerus.* N. B., aged 48: farmer. Fractured the right arm three inches above the elbow. I did not see him till several hours after the accident. Employed a long splint that extended three inches below the elbow and three inches above the shoulder, with holes in both ends. An axillary belt was used, and fastened through one of the holes at the upper end of the splint. Adhesive strips were passed spirally about the limb, and a loop formed at the elbow. Through this loop a cord was passed, and thence connected with one of the holes in the lower end of the splint. Extension and counter-extension were in this way procured. Circlets of adhesive strips two or three inches apart were used to confine the arm to the splint. The arm was flexed and a sling used.

CASE IV.—*Fracture of the Humerus.* A case similar to the above in many respects. Treated in the same way, except that a crutch-splint was put on the inside of the arm, its upper end padded by the axillary belt, and that tied over the shoulder. Extensive bruises on the arm necessitated this change of principles.

CASE V.—*Colle's Fracture.* A. B., shoemaker, in a drunken brawl fractured the left arm. A thin board, three inches wide, was used as a splint. This was placed on the posterior aspect of the forearm and provided with two compresses—one at the carpus and the other at the elbow. Adhesive strips were used to secure the splint in place, the application beginning at the elbow before the fracture was reduced. As soon as purchase was obtained there, the displaced parts were properly adjusted and adhesive strips applied at intervals from the elbow to the hand. Patient went on a prolonged spree a week after the accident, and exposure to a storm set up an excess of inflammation that delayed recovery. Usually in such cases I institute passive motion at the end of twenty days, repeating it daily for four weeks, when the splint is removed. I find it advisable in some instances to envelope the arm with bands of adhesive plaster after removing the splint. In this event the muscles act as splints, and take the place of the splint with the assistance of the adhesive strips.

CASE VI.—*Fracture of the Tibia just above the Ankle.* Thomas D., aged 30; mill operaive. Saw patient with Dr. L. a few minutes after the accident that resulted in the fracture. The bone was partially driven through the skin, and the fracture presented a complicated appearance.

*Treatment* as in Case II, except that it was necessary to elevate the limb by means of a cushion placed under the heel. The bed itself was better than all the patented splints in existence, as it kept the bone in place effectually. The limb was badly torn, and we found it advisable to cut the plasters several times the next day because of the pain and swelling. But new strips were supplied and the cord occasionally tightened until, on its perfect line of extension, the union of the osseous structure was complete.

CASE VII.—*Fracture of the Humerus at the Elbow.* This was a remarkable case. The patient, J. T., aged 18, was run over by a heavy wagon, the wheel crushing the elbow of the left arm. Examination showed that there was a fracture of both the forearm and humerus. The splint used was made "on the spur of the moment." Two strips of board, (shingles), one-fourth inch thick, and long enough when hinged by adhesive plasters to extend from the shoulder to two inches below the tips of the fingers, were used. It was fastened, while the arm was extended, by circlets of plasters, taking care to confine elbow closely. Forced flexion gave the required extension. I have never met with another case exactly like this. My practice is to have the hinge an inch above the elbow in a fracture of the forearm, and an inch below if the humerus is fractured. The plaster strips should be drawn as tight as possible and the forced flexion maintained.

Without any essential modification the principle of treatment in all these cases is that of Dr. Swinburne, whose description would be much more elaborate and lucid than is mine. In all that the phrase means this is simplicity in fracture-apparatus exemplified. With its employment I am glad to say my success is invariably excellent, all conditions being in ratio. The same cannot be said in my experience with any patented appliance. Bandages I rarely employ, as I do not consider that their use is always of advantage. They obstruct circulation, tend to create extra warmth, and prevent thorough examination. I always limit their use, and at the same time employ all of the adhesive plaster that is in any sense necessary. A clapboard, a shingle or a lath furnishes all the splints required; and any physician can find a suitable piece of board for his purpose.

Undeniably there are good manufactured splints, but the country practitioner, who needs all of his scanty earnings for bread and clothing, cannot afford them. Nor can he afford to be so retrograde as to carry an armful of shingles, as did the fathers. A roll of adhesive plaster, a knife and a good stock of wits is all that a physician needs to carry when summoned to attend a fracture case.

I would that the "Swinburne method" was better known. I would that simplicity was in the place of elaboration. The best measure of success has attended the use of these means, and the profession waits for the man who will in terse and vigorous language defend a course of treatment that he can ably describe, and which receives a blessing of benignant Nature.—*Louisville Med. News.*

